# Circulating levels of the adipocytokines vaspin and omentin in patients with Kawasaki disease

**DOI:** 10.1186/1546-0096-9-S1-P304

**Published:** 2011-09-14

**Authors:** L Cantarini, MG Brizi, G Simonini, T Giani, A Vitale, A Fioravanti, MR Bacarelli, M Galeazzi, R Cimaz

**Affiliations:** 1 Rheumatology Unit, Department of Clinical Medicine and Immunologic Sciences, University of Siena, Italy; 2 Department of Paediatrics, Rheumatology Unit, Anna Meyer Children's Hospital and University of Florence, Italy

## Background

Adipocytokines belong to a growing family of proteins mainly secreted by adipocytes which participate in a wide variety of physiological or physiopathological processes including immunity and inflammation. Vaspin and mentin are two recently discovered adipocytokines that have been involved in chronic inflammatory processes.

## Aim

Our aim was to evaluate serum omentin and vaspin levels in patients affected by complete Kawasaki disease (KD) in comparison to healthy controls.

## Methods

Our study group included 21 children (14 male, 7 female) with KD classified as complete on the basis of published standard clinical Criteria. The KD patients were enrolled within the 7th day from disease onset, with day 1 defined as the first day of fever symptoms. Serial blood samples were obtained from all KD patients in the acute phase before the IVIG therapy. Serum vaspin and omentin were analyzed by ELISA assays. All patients were administered both aspirin (30-50 mg/kg/day) and a single dose of intravenous immunoglobulins (IVIGs) (2 g/kg). Twenty-five healthy children (15 male, 10 female) were enrolled as controls.

## Results

Serum omentin levels were significantly higher in KD patients versus healthy controls (p< 0.0002); plasma vaspin levels did not significantly differ between the two groups (p =NS).

## Conclusion

Serum omentin levels are significantly elevated in acute KD in comparison to healthy controls. Omentin has recently been involved in chronic inflammatory processes and it can be hypothesized that its levels could be relevant to systemic inflammation and/or disease activity in KD.

